# Indole signaling in *Escherichia coli*: a target for antivirulence therapy?

**DOI:** 10.1080/19490976.2025.2499573

**Published:** 2025-05-07

**Authors:** Sofia Razzaq Meo, Tom Van de Wiele, Tom Defoirdt

**Affiliations:** Center for Microbial Ecology and Technology (CMET), Department of Biotechnology, Ghent University, Gent, Belgium

**Keywords:** Indole, quorum sensing, antivirulence therapy, virulence factors

## Abstract

Pathogenic *Escherichia coli* are a major cause of infections in both humans and animals, leading to conditions such as severe diarrheal diseases, urinary tract infections, enteritis, and septicemia. To combat bacterial infections, antibiotics are widely utilized. However, the extensive and inappropriate use of antibiotics has fueled the development and spread of antibiotic resistance, posing a significant challenge to the effective treatment of *E. coli*. There is consequently an urgent need to explore alternative therapies to control such infections. This review provides an overview of the recent findings concerning indole signaling in *E. coli*. *E. coli* uses indole as a quorum sensing molecule, and indole signaling has been reported to decrease various virulence factors in pathogenic *E. coli*, including motility, biofilm formation, adherence to host cells, expression of the LEE pathogenicity island, and formation of attaching and effacing lesions. This makes indole signaling an interesting target for the development of new therapeutics in the framework of antivirulence therapy. Both natural and synthetic indole analogues have been explored as potential virulence inhibitors. This alternative approach could be advantageous, as it will exert less selective pressure for resistance development than conventional antibiotics.

## Introduction

*Escherichia coli*, a Gram-negative bacillus, belongs to the family *Enterobacteriaceae* and resides in the gastrointestinal tract of humans and other warm-blooded animals, and contains several pathotypes.^1,2^ *E. coli* is among the first colonizing bacteria of the gut after birth. It is highly competitive and comprises the most abundant facultative anaerobe of the human intestinal microbiota.^3^ Being a facultative anaerobe, it can survive when released into the environment and subsequently spread to new hosts.^4,5^

Pathogenic *E. coli* are broadly separated into intraintestinal pathogenic *E. coli* (IPEC) responsible for intestinal infections, and extraintestinal pathogenic *E. coli* (ExPEC) which cause extraintestinal infections.^6^ Intestinal pathogens spread through the fecal-oral route by ingesting contaminated food or water.^1,7^ The treatment of infections caused by *E. coli* pathotypes frequently involves antimicrobials, thereby contributing to the emergence of antimicrobial resistance.

Antimicrobial resistance is a major and increasing global healthcare problem.^8^ Increased consumption and misuse of antimicrobial agents accelerate the prevalence of antimicrobial resistance.^9,10^ It is estimated that by 2050, drug-resistant bacterial infections will claim 10 million lives annually and result in a cumulative economic loss of $100 trillion. Among these, drug-resistant *E. coli* is projected to cause 3 million deaths and account for 40% of the economic impact.^6,11^ The issue of drug-resistant *E. coli* is causing significant alarm in human and veterinary medicine on a global scale. The 2022 Global Antimicrobial GResistance and Use Surveillance System (GLASS) report highlights significant resistance levels in *E. coli*. Among 76 reporting countries, the median resistance rate for the third-generation cephalosporin-resistant *E. coli* was 42%, emphasizing the global challenge posed by these resistant strains.^12^ Moreover, *E. coli* has been classified as a critical priority pathogen with respect to antimicrobial resistance by the World Health Organization (WHO) due to increasing antibiotic resistance,^13^ posing a significant threat to public health and highlighting the urgent need for developing new antimicrobial therapies.

One of the actions that has to be taken in order to control the alarming situation of antimicrobial resistance is the development of novel therapies. Antivirulence therapy, disarming rather than killing pathogens, has been suggested as an effective alternative to antibiotics.^14,15^ Antivirulence therapy aims to decrease the virulence of pathogens instead of killing them, by inhibiting the production of virulence factors (phenotypes required for infection). Bacterial cell-to-cell communication systems (quorum sensing) are amongst the targets of antivirulence therapy that have received the greatest attention.^16^ These systems control the virulence of various pathogens of plants, animals, and humans, and interfering with them can protect these organisms from disease. One of the quorum sensing molecules produced by *E. coli* is indole, and indole signaling has been documented to control various virulence factors in pathogenic *E. coli* and could therefore be an interesting target for the development of novel therapeutics. This review aims to address this possibility by synthesizing current knowledge on indole signaling in *E*. coli and exploring its potential as a therapeutic target. In this review paper, we first give a brief overview of the pathogenic *E. coli* types and then discuss the current knowledge with respect to indole signaling in *E. coli* and its interference.

## Brief overview of E. coli pathotypes

Pathogenic *E. coli* are significant causes of diarrheal diseases, particularly in vulnerable populations such as young children and elderly. Each pathotype utilizes distinct mechanisms to colonize the host, produce toxins, and cause intestinal or extraintestinal infections, ranging from watery diarrhea to life-threatening conditions like hemolytic uremic syndrome. Understanding the virulence factors and host interactions of these *E. coli* pathotypes is critical for developing effective prevention and treatment strategies, especially in areas with high infection rates. The diversity of *E. coli* is evident by varying colonization sites and pathologies caused by different pathotypes (Table 1).

**Table 1.** Intestinal human pathogenic *E. coli* pathotypes.

Pathotype	Host	Disease	Site of colonization	Virulence factors	References
Enteropathogenic *E. coli* (EPEC)	Children	Diarrhea	Small intestine	Intimin, LEE^a^	^17^
Enterohemorrhagic *E. coli* (EHEC)	Children and elderly people	Bloody, non-bloody diarrhea, hemolytic colitis, hemolytic uremic syndrome (HUS)	Terminal ileum, colon	Shiga toxin, intimin, LEE^a^	^18^
Enterotoxigenic *E. coli* (ETEC)	Children and adults	Watery diarrhea	Small intestine	Heat-labile and heat stable toxins, adhesins (fimbriae)	^19^
Enteroaggregative *E. coli* (EAEC)	Children and adults	Watery diarrhea, traveler’s diarrhea	Small intestine and/or colon	Fimbriae, cytotoxins	^20^
Enteroinvasive *E. coli* (EIEC)	Children	Watery diarrhea	Colon	Shiga toxin, hemolysin, Cellular invasion	^21^
Diffusely adherent *E. coli* (DAEC)	Children	Diarrhea	Intestine (Uncharacterized location)	Adhesins	^22^

^a^Locus of Enterocyte Effacement pathogenicity island.

Enteropathogenic *E. coli* (EPEC) are mainly responsible for diarrhea in young children because of their prevalence in the community as well as in hospital settings. EPEC cause nonspecific gastroenteritis. They are responsible for sporadic and epidemic outbreaks and are acquired by ingestion but can also spread from person to person.^23^ Approximately 10^8^–10^10^ EPEC bacteria are necessary to cause infection in adult humans.^24^ The plasmid pEAF encodes a bundle-forming pilus that helps EPEC to attach to enterocytes in the small intestine. Once bound, intimin, the outer membrane colonization factor, facilitates enhanced adherence. There are approximately 20 secretory toxins in the locus of enterocyte effacement (LEE) chromosomal island of EPEC that are injected into the intestinal cells by a type III secretion system.^17^

Enterohemorrhagic *E. coli* (EHEC) or Shiga toxin-producing *E. coli* (STEC) can cause bloody and non-bloody diarrhea, hemolytic colitis, and hemolytic uremic syndrome (HUS) in all age groups but particularly in the young and elder people and in immunocompromised patients.^25^ EHEC colonizes the human terminal ileum and colon and produces Shiga toxins Stx1 and Stx2 and infection is linked to raw dairy products and uncooked beef consumption.^18,26^ For EHEC to cause an infection, ingestion of 10–100 cells is needed.^24^ The Shiga toxins are a group of bacterial binary toxins consisting of one A subunit that is responsible for inhibition of protein synthesis and five identical B subunits that bind with the glycosphingolipid Gb3, a cellular receptor on endothelial cells. The inhibition of protein synthesis leads to intestinal cell death and subsequent inflammatory colitis.^27^ In addition to Shiga toxin production, EHEC expresses a type III secretion system (T3SS), a specialized syringe-like apparatus that translocates bacterial effector proteins directly into the host cells, enabling the bacteria either to mimic or subvert the host cell function.^1^ The EHEC T3SS, encoded on the locus of enterocyte effacement (LEE), a pathogenicity island, is essential for intestinal colonization and disease development.^28^ Effector proteins injected through the translocon interact with host proteins, triggering actin polymerization and formation of attaching and effacing (AE) lesions, which promotes tight bacterial adherence to host cells and leads to effacement of intestinal microvilli.^29^

Enterotoxigenic *E. coli* (ETEC) adheres and colonizes the small intestine with the help of colonization factors (CFs) or coli surface antigens (CSs) composed of one or two repeating subunits organized into fimbrial, fibrillar or afimbrial structures.^30^ Over 20 CF variants have been identified, most designated as “CS” followed by a number reflecting their order of discovery.^31^ ETEC produces plasmid-encoded heat labile (LT) and heat stable (ST) enterotoxins.^19^ However, the toxin is retained within the periplasm until ETEC arrives at its specific alkaline niche of infection adjacent to the epithelium in the small intestine.^32^ Once ETEC have adhered, toxins are secreted, which stimulate adenylate cyclase and guanosine cyclase, respectively. The former causes an increase in intracellular cyclic adenosine monophosphate (cAMP) while the latter enhances intracellular guanosine monophosphate, both resulting in subsequent chloride secretion and inhibition of sodium chloride absorption, thus producing watery diarrhea.^33^ The infectious dose range for ETEC is 10^8^-10^10^.

Enteroaggregative *E. coli* (EAEC) cause watery diarrhea in children and traveler’s diarrhea in both children and adults in resource limited and resource rich regions. The site of colonization for EAEC is the intestinal mucosa (terminal ileum) and predominantly that of the colon.^20^ EAEC exhibits a characteristic aggregative adhesion (AA) pattern, which results in ‘stacked bricks’-like bacterial aggregates adhering to epithelial cells.^34^ Aggregative adherence fimbriae (AAF) are essential for EAEC adherence to epithelial Hep-2 cells and other surfaces. Five genetically distinct AAF types (AAF/I to AAF/V) have been identified.^35^ A typical AAF operon encodes four genes: *aggD* (chaperone), *aggC* (usher), *aggB* (minor pilin), and *aggA* (major pilin). EAEC exhibits abundant adherence to intestinal mucosa, accompanied by the formation of mucoid biofilms, a key feature contributing to its pathogenicity. Deletion of the major pilin gene or entire AAF operon leads to reduced biofilm formation for AAF/I to AAF/V strains.^36^ In adults, an infectious dose of > 10^8^ CFU is needed.^37^

Enteroinvasive *E. coli* (EIEC) is an enteritis-causing pathogen and causes invasion of the large bowel resulting in inflammation and ulceration of the mucosa. Its occurrence is limited because a relatively large infective dose i.e. 10^6^-10^10^ EIEC is required.^21,38^ Subsequent colonization and invasion of colonic mucosa, replication, and cell-to-cell spread result in inflammatory colitis.^1^

Diffusely adherent *E. coli* (DAEC) are characterized by the presence of a diffuse adherence pattern that comprises bacteria that are evenly distributed over the complete surface of epithelial cells. There is no report for the infectious dose for DAEC. DAEC causes acute diarrhea in children.^22^ Extraintestinal infections are normally caused by the translocation of the *E. coli* outside the intestine.

In addition to the virulence factors that are typically associated with the specific *E. coli* pathotypes, there are additional virulence factors that are shared by all pathotypes (and other pathogens). These include flagellar motility and chemotaxis, as well as extracellular polysaccharide production and biofilm formation.^39,40^ Interestingly, all of the above-mentioned pathotypes are capable of producing indole.^41^ Thus, interfering with the indole signaling might open new possibilities for antivirulence therapy targeting *E. coli* pathotypes.

## Factors influencing indole production by E. coli

Indole is a heterocyclic molecule that is produced by the tryptophanase enzyme (TnaA) from the amino acid L-tryptophan.^42^ It has been reported that in LB medium, *E. coli* K-12 produces indole until a final concentration of approximately 0.5–0.6 mM.^43^ The highest production of indole occurs when the cells approach stationary phase, and this coincides with an upregulation of *tnaA* expression.^44^ Upon stationary phase entry, the cell-associated indole reaches a very high concentration (up to 60 mM) during a short period (10–15 min).^45^ It has been argued that this indole pulse inhibits growth of *E. coli* and causes cells to enter stationary phase before resources are exhausted, which enables the cells to survive starvation.

TnaA is part of the tryptophanase operon, which contains the genes involved in tryptophan metabolism and indole production. *tnaB* encodes a tryptophan specific permease.^46^ TnaC is a regulatory protein that controls the expression of the *tna* operon.^47^ TrpE is a component of the tryptophan synthase enzyme complex, which is responsible for the biosynthesis of tryptophan.

Indole production is dependent on the presence of tryptophan. An increase in the tryptophan concentration from 1 mM to 5 mM increases indole production (up to 4.7 mM), but   further addition of tryptophan does not further increase indole production even after extended incubation, suggesting that this is the maximum level of indole that cells can produce.^48^ Glucose has been reported to repress the indole biosynthesis by inhibiting *tnaA* transcription via catabolite repression.^42^ Glucose, at a concentration of 2 g/L, inhibits indole production and suppresses *tnaA* expression.^45^ A decrease in extracellular indole in *E. coli* K-12 cultures from 450 µM ±70 µM to 15 µM ±6 µM has been reported when glucose was added to the medium.^49^ Indole is also produced in the colon in mammals, where levels up to 1.1 mM have been reported.^50,51^ As indole is produced from the amino acid L-tryptophan by the tryptophanase enzyme in a reaction that also produces pyruvate (which can feed the TCA cycle to generate energy), it can serve as an indicator for protein fermentation (i.e. the generation of energy from protein). This mainly occurs toward the distal regions of the mammalian gastrointestinal tract.^52^ In contrast, carbohydrate fermentation occurs in the upper part of the gastrointestinal tract, and indole production will be inhibited there by catabolite repression. Recently, it was shown that indole is produced in the lumen of the large intestine by the gut microbes^53^ and uptake by the intestinal epithelial cells results in a higher concentration of indole in the lumen of the colon than in proximity of the colonic tissues.^54^ Hence, as *E. coli* passes through the intestinal tract, it can use indole as a cue informing the bacterium about its position in the intestinal tract.

Various environmental factors such as pH, temperature, and the presence of specific enzymes and antibiotics modulate indole production, highlighting its dynamic role in bacterial adaptation. *E. coli* increases *tnaA* expression at an alkaline pH.^55^ When the pH is alkaline, indole levels of 400 µM were reported in *E. coli* K-12, but with an acidic pH, there was a decrease in indole production to 100 µM.^56,57^

*E*. *coli* has been reported to utilize indole signaling in different ways depending on the temperature. Indole signaling affecting biofilm formation, cell division, and antibiotic resistance in *E. coli* occurs primarily at low temperatures (25–30°C), while control of plasmid stability happens at high temperature (37°C).^58,59^

In the presence of antibiotics such as ampicillin and kanamycin, *E. coli* K-12 produces a high level of indole, i.e. 1900 ± 260 µM indole/OD_600_ in the presence of ampicillin and 338 ± 87 µM indole/OD_600_ in the presence of kanamycin, vs. 88 ± 3 µM indole/OD_600_ without antibiotics. Further, a 2- to 5-fold increase in cell growth has been observed in the presence of ampicillin when exogenous indole (1 mM) was added to either wild type *E. coli* K-12 or a *tnaA* deletion mutant. Other antibiotics (e.g. chloramphenicol), in contrast, have been reported not to affect indole production in *E. coli* K-12, indicating that the effect varies between different antibiotics.^56,59^

The impact of other environmental factors, including osmolarity, aeration, heavy metals, and oxidative stress, on indole production in *E. coli* has also been investigated. However, none of these affected the indole production in *E. coli*.^56^

## Proteins involved in the response to indole in E. coli

**Figure 1.**
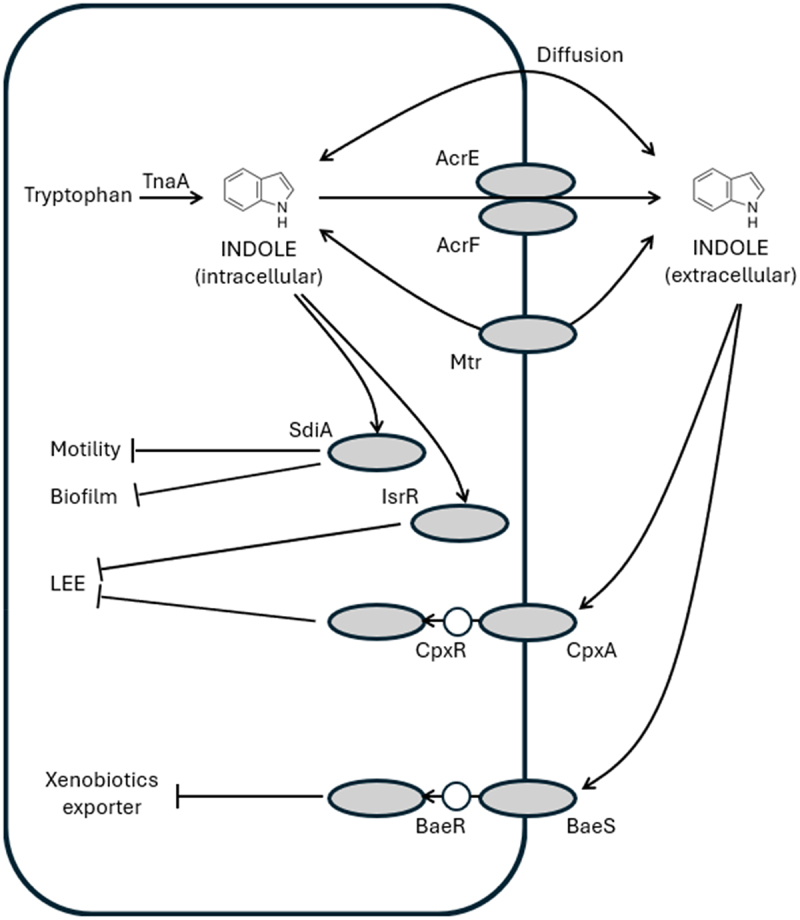
Mechanisms of indole production and indole-mediated regulation in *E. coli*. The white circles indicate phosphotransfer. See text for a detailed explanation.

In order to act as an extracellular signaling molecule, indole needs to leave the producing bacterial cells (Figure 1). At this moment, there is no consensus on how indole is transported across the cell membrane. On the one hand, protein-mediated transport through Mtr and AcrEF has been proposed,^60^ while on the other hand, it has been shown that indole can rapidly cross the *E. coli* cell membrane without the aid of a transport protein.^61^

Although indole has been documented to affect many phenotypes and to regulate numerous genes in *E. coli* (see below), the identification of an indole receptor has been more problematic, and thus far, binding of indole to any protein has not been demonstrated. One protein that has been implicated in sensing of indole, however, is the orphan acylhomoserine lactone receptor SdiA.^62^ *SdiA* was one of the most-highly induced genes in *E. coli* K-12 upon addition of 600 µM indole, and SdiA-mediated transcription was shown to be influenced by indole. However, *sdiA* deletion did not abolish all responses to indole, indicating that other factors are involved in the response to indole. Apart from SdiA, also the two-component systems BaeSR and CpxAR have been implicated in indole sensing in *E. coli*.^63^ The authors suggested that BaeR is a primary regulator, while CpxR enhances the effect of BaeR. Recently,^54^ confirmed the role of CpxA as sensor of exogenous indole in EHEC. The CpxAR system was the most significantly regulated two-component system by indole, with the expression of *cpxA* being decreased by exogenous indole, and a *cpxA* deletion mutant not responding to indole.

More recently,^64^ reported that another response regulator, IsrR, is involved in the response of EHEC to endogenous indole, as the bacterium still responded to exogenous indole independently from IsrR. The authors argued that IsrR aids in discriminating between exogenous (microbiota-derived) and endogenous (self-produced) indole.

Finally, the stationary phase sigma factor RpoS, the AraC type transcription factor GadX and the regulator Hfq have also been documented to be required for the response of *E. coli* to indole.^65^

## Phenotypes controlled by indole signaling in E. coli

High concentrations of indole (3–5 mM) block *E. coli* K-12 cell division due to its activity as a proton ionophore, reducing the electrical potential across the cytoplasmic membrane.^66,67^ Recently, it has been shown that this effect remains as a “memory” to maintain the cytoplasmic pH (Zarkan et al., ^68^2019). Lower concentrations of indole (≤1 mM), in contrast, do not affect viability of *E. coli*, but affect various phenotypes (Table 2), specified in the following paragraphs.

**Table 2.** Phenotypes affected by indole in *E. coli.*

Phenotype	Effect of indole	Concentration (µM)	*E. coli* type	References
Biofilm formation	Decrease	500–1000	K-12, EHEC	^69–71^
Motility	Decrease	500	K-12, EHEC	^62,70^
Adherence to host cells	Decrease	500	EHEC	^70^
LEE gene expression	Decrease	250	EHEC	^72–74^
	Increase	N/A^a^	EPEC	
Formation of AE lesions	Decrease	500	EHEC	^54^
	Increase	N/A^a^	EPEC	^75^
Virulence to a host	Increase	N/A^a^	EPEC	^75^
Acid tolerance	Increase	2000	K-12	^62^
Heat resistance	Increase	N/A^a^	K-12	^76^
Antibiotic resistance	Increase	200–2000	K-12	^77,78^
Persister cell formation	Increase	500	K-12	^79,80^
	Decrease	500–2000	K-12	^81^

^a^comparison between wild type and *tnaA* deletion mutant.

### Biofilm formation

A biofilm develops when a community of bacteria is surrounded by self-produced polymeric substances, often adhered to a surface. Biofilm bacteria exhibit distinctive phenotypes when compared to planktonic cells. Indole has been shown to decrease the biofilm formation in *E. coli* K-12. It was also reported that the effect of indole on the biofilm formation was temperature-dependent. When exposed to 1 mM indole, a 16- and 7-fold reduction in *E. coli* K-12 biofilm formation was observed at 25°C and 30°C, respectively.^59^ *trpE* and *tnaC* deletion mutant strains of *E. coli* K-12 (both show decreased indole production) produced 5.4- and 3.9-fold increased biofilm levels, respectively, compared to wild-type *E. coli* K-12. The addition of 500 µM and 1000 µM indole to the mutant strains decreased biofilm formation in a dose-dependent manner.^62^ Finally, the addition of 500 µM indole resulted in a 2.4-fold decrease in biofilm formation of EHEC.^70^

### Flagellar motility and chemotaxis

Indole has been reported to decrease the motility in *E. coli* K-12: the addition of 500 µM indole resulted in a 30–40% decrease in motility.^62^ Moreover, the addition of 500 µM indole also resulted in a 2.8-fold decrease in motility of EHEC.^70^ Further, when *E. coli* K-12 was exposed to low or high concentrations of indole (20 µM vs.1 mM or more), repellent and attractant responses were observed, respectively, which were mediated by the Tsr and Tar chemoreceptors.^82^

### Expression of the locus of enterocyte effacement (LEE) and adherence to host cells

It has been reported that indole (at 500 µM) decreased the adherence of EHEC to HeLa cells by 3.1-fold. Further, indole at 500 µM decreases the expression of genes involved in biosynthesis of flagella, chemotaxis, and flagellar motor activity. Thus, in the presence of indole, the decrease in the number of flagella causes a decrease in the motility of EHEC.^70^

Further, indole at 125 µM has been found to increase the secretion of type III secretion system proteins that are involved in the pathogenicity of EHEC strain Sakai (RIMD 0509952), and also increased the subsequent formation of attaching and effacing lesions.^72^ In contrast, indole, at increasing concentrations (ranging from 1 µM to 250 µM) resulted in a decrease in LEE gene expression in EHEC 86–24 strain (including type III secretion system genes). Further, the expression of the LEE encoded genes (*espA, tir, eae*), and the non-LEE encoded Shiga toxin gene (*stx2a*) was higher in ∆*tnaA* EHEC than in the wild type, whereas treatment of the ∆*tnaA* mutant with indole decreased the expression of these genes again.^54,73^ Finally, attaching and effacing lesion formation was enhanced in the ∆*tnaA* mutant strain when compared to the wild type EHEC, and addition of 500 µM indole decreased lesion formation. In contrast to the above,^75^ reported that tryptophanase activity was required for full activation of the LEE1 promoter and for the formation of attaching and effacing lesions by EPEC.

### Virulence of pathogenic E. coli to a host

Few studies have determined the impact of indole signaling on the virulence of pathogenic *E. coli* in animal models.^75^ found that a *tnaA* deletion mutant of EPEC lost its virulence in the nematode *Caenorhabditis elegans*. The authors did not elucidate, however, whether the effect was due to the lack of indole or to another effect caused by *tnaA* deletion. Recently,^54^ reported that microbiota-produced indole decreased the virulence of *Citrobacter rodentium*, which is used as a murine model for EHEC infections (given that EHEC is not pathogenic to mice).

### Resistance to stress

The acid resistance genes *gadABC* that are regulated by GadE, protect and help *E. coli* to survive at acidic pH, e.g. in the stomach.^83^ Indole has been reported to decrease expression of these acid resistance genes by 2- to 4-fold. The addition of 2 mM indole to wild-type *E. coli* K-12 resulted in 350- to 650-fold decreased survival at pH 2.5.^62^ Indole has further been documented to protect *E. coli* K-12 cells from heat stress during exponential growth. Although the survival rate was similar for both wild-type and ∆*tnaA* (indole deficient) *E. coli* after 2 hours at 50°C, wild-type cells showed a 10-fold higher survival rate after 24 hours.^76^

### Antibiotic resistance and tolerance

Indole has been reported to induce antibiotic resistance in *E. coli*. Lee et al.^78^ reported that indole, at 200 µM, upregulated multi-drug efflux pumps such as *mdtE* in *E. coli* K-12. They further documented that indole production by highly resistant mutants in this way protected the whole *E. coli* population from the quinolone norfloxacin, and enabled the *E. coli* population to grow in the presence of increasing concentrations of the antibiotic (up to 2.5 mg/L) in a continuous bioreactor.

Further, it has been reported that indole induces the overexpression of the multidrug exporter genes *acrD, acrE, cusB, emrK, mdtA, mdtE* and *yceL* in *E. coli* K-12. Further, the addition of indole enhanced survival of *E. coli* with 60% and 4.5% when treated with 12.5 mg/L rhodamine 6 G or 100 mg/L SDS, respectively. The deletion of *mdtEF* decreased the indole-mediated survival to rhodamine 6 G to 15%, and *acrD* deletion resulted in complete loss of indole-induced SDS resistance.^63^ It should be noted, however, that the induction of the multidrug exporter genes occurred at relatively high (≥1 mM) concentrations of indole.

Temperature has been documented to affect the efficiency of indole in modulating the antibiotic tolerance in *E. coli* K-12. The addition of 1 mM indole to a *tnaA* deletion mutant treated with 0.1 mg/ml kanamycin resulted in a 30-fold increased survival at 30°C, whereas at 37°C, the increase in survival was only twofold.^59^

### Persister cell formation

Persisters are a sub-population of genetically sensitive bacteria that survive antibiotic treatment by entering a dormant state.^80^ After the withdrawal of the antibiotic, these dormant cells can reactivate their metabolism, leading to recurrent infection. Indole has been found to stimulate the formation of *E. coli* persisters against quinolones. When exposed to 100 × MIC of quinolones, more persisters were observed in wild-type *E. coli* K-12 cultures than in cultures of the indole non-producing *tnaA* deletion mutant. The effect was small (i.e. 2- to 4-fold) for nalidixic acid, levofloxacin, and moxifloxacin, but was high (i.e. 10-fold) for ciprofloxacin.^80^ Wild-type *E. coli* K-12 produced at least an order of magnitude more persisters in the presence of 5 µg/ml ofloxacin, 100 µg/ml ampicillin or 10 µg/ml kanamycin, when they were pre-incubated with 500 µM indole. Similarly, *tnaA* deletion decreased the persister formation of *E. coli* K-12 by almost 10-fold in comparison to the wild-type. Further, the addition of 500 µM indole to the *tnaA* mutant increased the persister formation again, suggesting that the effect of *tnaA* deletion on persister levels was mediated by indole.^79^

Others have documented, however, that indole decreases persistence,^84^ and this was linked to the toxin/antitoxin system YafQ/DinJ.^81^ Production of the toxin YafQ increased persister formation in *E. coli* K-12 with ampicillin or ciprofloxacin, whereas it decreased tryptophanase levels, and the addition of indole (0.5–2 mM) decreased persistence of a *tnaA* deletion mutant. It was further shown that DosP (direct oxygen sensing phosphodiesterase) increases persistence of *E. coli* K-12 by decreasing tryptophanase activity^85^ Despite the role of DosP as a c-di-GMP phosphodiesterase, the decrease in tryptophanase activity was found to be a result of cyclic adenosine monophosphate (cAMP) phosphodiesterase activity.

### Interaction of E. coli with other microbes

It has been suggested that indole can facilitate the competitiveness of *E. coli* in mixed cultures.^86^ Indeed, a *tnaA* deletion mutant of *E. coli* K-12 was less competitive than the wild type in both planktonic and biofilm mixed cultures with *Pseudomonas aeruginosa*, and the addition of 1 mM indole restored the competitiveness.^87^ Another study has shown that indole can also have a negative effect on *E. coli* competitiveness in mixed cultures. Indeed, a *tnaA* deletion mutant of *E. coli* K-12 showed a higher fitness than the wild type in coculture with *Enterococcus faecalis*, and supplementation of indole removed the competitive advantage of the *tnaA* deletion mutant in planktonic co-cultures, but enhanced it in biofilm co-cultures.^88^ Therefore, the impact of indole on the competitiveness of *E. coli* is complex and can vary depending on the specific microbes in its environment. Further research is needed to fully understand the role of indole in shaping microbial communities.

## Impact of indole analogues on E. coli

**Figure 2.**
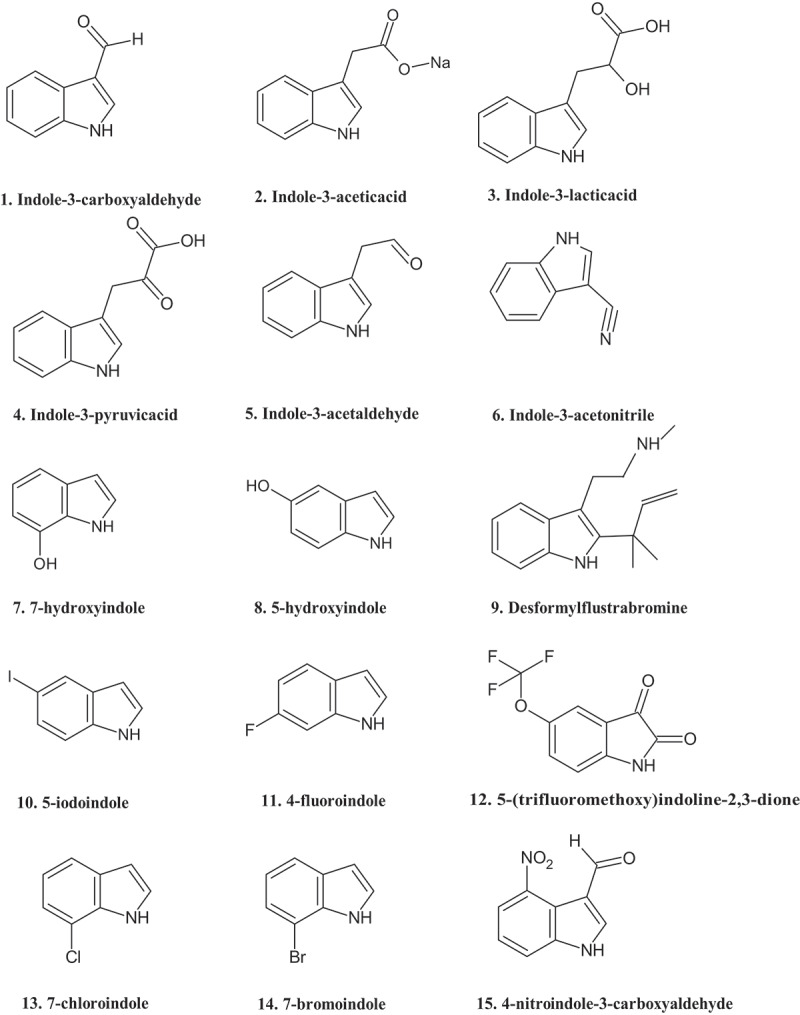
Structure of selected indole analogues that were tested in *E. coli.*

Because of the impact of indole on pathogenic *E. coli*, several authors studied indole analogues in order to identify more stable and more potent virulence inhibitors (Figure 2 ; Table 3). Like indole, indole derivatives can regulate different cellular processes in *E. coli*. Some indole analogues, i.e. indole-3-carboxyaldehyde and indole-3-acetic acid (at concentrations >1 mM) were found to activate the expression of multiple genes such as those involved in stress response, antibiotic resistance, and virulence in EHEC, EPEC, and EAEC.^90^

**Table 3.** Impact of indole analogues on *E. coli.*

Compound	Concentration (µM)	*E. coli* type	Effect	References
Indole-3-lactic acid, indole-3-pyruvic acid and indole-3-acetaldehyde	488, 493 and 629	EHEC	Decreased biofilm formation	Lee et al.^89^
Indole-3-carboxyaldehyde	1500	EPEC	Decreased pedestal formation	Bommarius et al.^90^
	>1000	EHEC, EPEC, EAEC	Increased expression of genes involved in stress response, antibiotic resistance, and virulence	Bommarius et al.^90^
	690	EHEC	Decreased biofilm formation	Lee et al.^91^
	1500	EHEC, EAEC	Decreased cytotoxicity	Bommarius et al.^90^
Indole-3-acetic acid	>1000	EHEC, EPEC, EAEC	Increased expression of genes involved in stress response, antibiotic resistance, and virulence	Bommarius et al.^90^
	571	EHEC	Decreased biofilm formation	Lee et al.^89^
	500	K12	Increased stress survival	Bianco et al.^92^
Halogenated indoles	2000	K12	Decreased survival of persister cells	Lee et al.^93^
Desformylflustrabromine analogue	100	K12	Decreased biofilm formation	Bunders et al.^94^
7-hydroxyindole and 5-hydroxyindole	500	EHEC, K12	Decreased biofilm formation	Lee et al.^71^
4-nitroindole-3-carboxyaldehyde	1500	EHEC	Decreased cytotoxicity	Bommarius et al.^90^
3-indolylacetonitrile	641	EHEC	Decreased biofilm formation	Lee et al.^91^

The indole analogues indole-3-acetic acid, indole-3-lactic acid, indole-3-pyruvic acid and indole-3-acetaldehyde (at 100 µg/ml) have been reported to decrease biofilm formation in EHEC.^89^ Indole-3-carboxyaldehyde and 3-indolylacetonitrile have been reported to inhibit biofilm formation in EHEC by 11-fold and 24-fold, respectively, at 100 mg/L. 3-indolylacetonitrile was eight times more potent as biofilm inhibitor than indole in EHEC at 37°C.^91^ Further, 7-hydroxyindole at 500 µM decreased EHEC and *E. coli* K-12 biofilm formation by 27-fold and 8-fold, respectively, while 5-hydroxyindole decreased biofilm formation with 11-fold and 6-fold for the respective bacteria. A desformylflustrabromine analogue, at 100 µM, decreased biofilm formation of *E. coli* K-12 with 82%.^94^

Various halogenated indole derivatives were found to affect the survival of persister cells of *E. coli* K-12. At 2 mm, 5-iodoindole, 4-fluoroindole, 5-(trifluoromethoxy)indoline-2,3-dione, 7-chloroindole and 7-bromoindole decreased persister survival by more than 2000-fold.^93^ Indole derivatives have also been documented to downregulate the production of LEE virulence factors and inhibit pedestal formation. Indole-3-carboxyaldehyde decreased the pedestal formed by EPEC on 3T3 cells by 7-fold at 1.5 mM.^90^ Further, indole-3-carboxyaldehyde (at 1.5 mm) decreased the IC_50_ for the cytotoxicity of Shiga toxins produced by EHEC and EAEC by 10-fold, while 4-nitroindole-3-carboxyaldehyde (at 1.5 mM) caused a 100-fold reduction in the IC_50_ for Shiga toxin cytotoxicity in EHEC.^90^

Indole-3-acetic acid, at 500 µM, has been found to improve the defenses to stress in *E. coli* K-12. The survival percentage for acid shock (pH 3), osmotic shock (0.5 M NaCl), UV irradiation (100 J/m^2^), heat shock (55°C) and oxidative stress (2 mm H_2_O_2_) in the presence of indole-3-acetic acid have been reported to be 81 ± 2, 72 ± 1, 92 ± 7, 21.5 ± 5, and 97 ± 6, respectively, compared to 46 ± 5, 52 ± 1, 51 ± 4, 1.7 ± 0.3, and 56 ± 2 for untreated *E. coli* K-12.^92^

## Conclusions and further perspectives

Pathogenic *E. coli* types are significant contributors to human disease. The widespread and improper use of antibiotics has resulted in the global development of antibiotic resistance, making *E. coli* a critical priority pathogen with respect to antibiotic resistance. It is estimated that by 2050, out of 10 million deaths/year due to drug-resistant pathogens, 3 million will be only projected to drug-resistant *E. coli*. To overcome this societal challenge, there is a need to develop alternative therapies. Antivirulence therapy, which aims at disarming pathogens instead of killing them, is gaining interest as a novel strategy to control bacterial infections. Antivirulence therapy relies on disruption of quorum sensing systems, bacterial cell-to-cell communication or other virulence factors. One of the quorum-sensing molecules that *E. coli* produces is indole, and indole signaling has been documented in pathogenic *E. coli* types (mainly EHEC) to interfere with virulence factors such as biofilm formation, motility, adherence to host cells, expression of the LEE pathogenicity island and formation of AE lesions. It will be interesting to determine the impact of indole signaling on other pathogenic types of *E. coli*, and to determine the impact of indole on virulence of pathogenic *E. coli* in animal infection models. This will reveal whether indole signaling indeed is an effective target for antivirulence therapy against different pathogenic *E. coli*. By targeting virulence rather than viability, indole could reduce the selective pressure for resistance development, offering an advantage over conventional antimicrobials. In addition to this, experiments with infection models will reveal whether indole itself is useful as virulence inhibitor, or whether it would be better to look for more potent and/or less toxic analogues. Preliminary results indicated that such compounds can indeed be identified. Finally, farmaceutical administration modes of the virulence inhibitors will need to be investigated.
